# Tau positron emission tomography results from TOGETHER, a double‐blind, placebo‐controlled Phase II study of bepranemab in prodromal–mild Alzheimer’s disease

**DOI:** 10.1002/alz70859_099279

**Published:** 2025-12-25

**Authors:** Ralph P Maguire, William Byrnes, Matthew E Barton, Irene Rebollo Mesa, Tim J Buchanan, Bart Van Den Steen, Hans L.G Van Tricht, Katie Taiyari, Omobola Famodimu, Jos Bloemers, Steve Einstein, Colin Ewen

**Affiliations:** ^1^ UCB, Braine‐l’Alleud, Walloon Brabant Belgium; ^2^ UCB, Raleigh, NC USA; ^3^ UCB, Madrid, Madrid provincia Spain; ^4^ UCB, Slough, Berkshire United Kingdom; ^5^ UCB, Oakville, ON Canada; ^6^ UCB, Breda, North Brabant Netherlands; ^7^ UCB (contracted via Steve Einstein Consulting Services, Lawrenceville, New Jersey, USA), Braine‐l’Alleud, Walloon Brabant Belgium

## Abstract

**Background:**

Bepranemab is a recombinant, humanized, full‐length immunoglobulin G4 monoclonal antibody that targets a mid‐region epitope of human tau. TOGETHER (NCT04867616), a Phase II participant‐ and investigator‐blinded, randomized, placebo‐controlled study, assessed efficacy and safety of bepranemab in people with prodromal–mild Alzheimer’s disease (AD). Previously presented data from TOGETHER indicate a clinical benefit with bepranemab. Here, we describe the effect of bepranemab on tau accumulation in the brain in study participants with prodromal or mild AD.

**Methods:**

Participants (aged 50–80 years) received bepranemab (45 mg/kg or 90 mg/kg, intravenously every 4 weeks) or placebo over an 80‐week treatment period. Participants received [^18^F] Genentech tau probe 1 (GTP1) and underwent positron emission tomography (PET) imaging at Baseline, Week 56, and Week 80. Eligibility criteria included prodromal–mild AD (National Institute on Aging and the Alzheimer's Association 2018 Stage 3/4); cerebral amyloid beta presence (PET or cerebrospinal fluid); global Clinical Dementia Rating (CDR) score of 0.5; CDR‐Memory Box score ≥0.5. Secondary objectives included investigating the effect of bepranemab on tau‐PET imaging at Weeks 56 and 80. PET images were analyzed by a central imaging laboratory to determine the standardized uptake value ratio relative to cerebellum in multiple brain regions.

**Results:**

In total, 466 participants were randomized 1:1:1 across three arms (90 mg/kg bepranemab, 45 mg/kg bepranemab, placebo). Bepranemab slowed tau accumulation (by 33–58% vs placebo, across both bepranemab arms) in the whole cortical gray (n=scanned/total: 90 mg/kg bepranemab [n=113/152], 45 mg/kg bepranemab [n=104/152], and placebo [n=97/156]) and jack temporal meta regions (n=scanned/total: 90 mg/kg bepranemab [n=114/152], 45 mg/kg bepranemab [n=105/152], and placebo [n=97/156]) at Week 80 in the initial analysis of the full trial population (those who received at least a partial dose of study medication and had at least one valid post‐Baseline clinic visit and efficacy assessment). Data on tau accumulation in regions based on Braak staging will be presented.

**Conclusion:**

TOGETHER provides the first clinical demonstration of slowing of tau accumulation with an antibody targeting the tau mid‐region, as evidenced by tau PET imaging, and marks the first time that any tau‐directed therapy has demonstrated a clinical benefit.

